# Two-dimensional vacancy-doped MXene nanomaterials for supercapacitors

**DOI:** 10.3389/fchem.2025.1656521

**Published:** 2025-07-23

**Authors:** Yi Tang, Zhao Bi, Yangyang Xie, Xiaodie Xuan, Chenhui Yang

**Affiliations:** ^1^College of Materials Science and Engineering, Xi’an University of Science and Technology, Xi’an, Shaanxi, China; ^2^School of Chemistry and Chemical Engineering, Northwestern Polytechnical University, Xi’an, Shaanxi, China

**Keywords:** 2D materials, MXene, vacancy doping, supercapacitors, energy storage mechanisms

## Abstract

Supercapacitors (SCs) are high-performance electrochemical energy storage devices, and their performance hinges on the electrode materials. 2D MXene nanomaterials, with their excellent conductivity, tunable interlayer spacing, and rich surface chemistry, have emerged as highly promising electrode materials for SCs. However, the capacitive performance of intrinsic MXene fails to meet application requirements. This review first introduces the composition and principles of SCs in detail, then summarizes the pure MXene nanomaterials in SCs, and systematically explores the regulatory mechanisms of vacancy doping strategies on MXene material structure and capacitive performance. The study reveals the structure-property relationships, providing theoretical basis and direction for designing high-performance MXene-based SCs electrode materials.

## 1 Introduction

With their outstanding power density and fast charging and discharging capabilities, supercapacitors (SCs) have become important energy storage components in key areas such as electric vehicles (regenerative braking systems), renewable energy grid connection (power fluctuation smoothing), smart grids (fast frequency response), and wearable/flexible electronic devices. As a new type of energy storage device that bridges the gap between traditional capacitors and batteries (Janardhanan et al., 2025), SCs are considered promising electrochemical energy storage devices due to their unique performance and advantages, including ultra-high power density (Yadav and Srivastava, 2025), extremely long cycle life (Zhao et al., 2025), rapid charging and discharging capabilities ranging from seconds to minutes (Lee et al., 2025), high safety (Wang et al., 2018), and a wide temperature range (Xu et al., 2025). Generally speaking, based on two energy storage mechanisms: electric double-layer capacitance achieved through ion adsorption and pseudocapacitance achieved through rapid surface redox reactions between the electrolyte and electrode surface. SCs comprise three types based on charge storage: (i) Electric double-layer capacitors (EDLCs) store charge electrostatically at the electrode-electrolyte interface, offering high power density and long cycle life (Shen et al., 2024); (ii) Pseudocapacitors (PCs) utilize fast, reversible surface redox reactions for higher energy density (Cui et al., 2024); (iii) Hybrid supercapacitors (HSCs) combine EDLC and PC mechanisms/electrodes to balance performance (Gharanli et al., 2025; Xuan et al., 2025).

For EDLCs, when electrode materials (such as activated carbon, carbon fiber, etc.) are immersed in an electrolyte containing ions, the charges on the electrode surface attract ions of opposite charge in the electrolyte, thereby forming a double-layer structure with charge separation between the electrode and the electrolyte (Atlas and Ramon, 2018). Due to its rapid and reversible charge adsorption process, which does not involve redox reactions, it has excellent stability and high-power characteristics. However, its precisely this essentially purely electrostatic charge storage mechanism limits the energy density (Shen et al., 2024). In contrast, pseudocapacitive energy storage of PCs relies on rapid redox reactions, which primarily occur at the electrode/electrolyte interface and its near-surface regions. These reactions typically depend on conductive materials with high specific surface areas and abundant electrochemical active sites, such as transition metal oxides or conductive polymers. These active sites facilitate the rapid reversible accumulation and release of charge (ions/electrons) on the electrode surface, significantly enhancing the device’s charge storage capacity and thereby exhibiting higher energy density and specific capacitance (Xu et al., 2022). However, due to the Faraday effect, PCs typically have lower power than EDLCs (Chuang et al., 2010). HSCs combine double-layer and pseudocapacitive mechanisms, increasing energy density by introducing pseudocapacitive materials while maintaining high power output and fast charging/discharging characteristics of carbon materials (Cheng et al., 2021). Therefore, it is important to develop advanced SCs by constructing electrode materials with double-layer and pseudocapacitive behaviors.

Researchers are continuously exploring new electrode materials, such as metal oxides (Orera et al., 2022), metal sulphides (Oh et al., 2017), metal carbides (Sheikh et al., 2024), metal nitrides (Adalati et al., 2022), metal hydroxides (Gonçalves et al., 2020), metal-organic frameworks (MOFs) (Sarac et al., 2025), and MXene (Jiang et al., 2020), to further enhance the overall performance of SCs, particularly in terms of increasing energy density. Among them, MXenes, two-dimensional transition metal carbides, nitrides, and carbonitrides, are a novel nanomaterial with a structure similar to graphene, prepared by selectively etching its precursor MAX phase (i.e., M_*n*+1_AX_*n*_, where M is an early transition metal, A is a Group III or IV element, X is C or N, and n = 1, 2, 3) (Wang et al., 2023; He et al., 2024). Since Michael Naguib et al. first discovered Ti_3_C_2_T_*x*_ in 2011 (Lukatskaya et al., 2013), exceed 30 MXene variants with different compositions have been successfully synthesized, and over 100 MXene configurations have been theoretically predicted (Zhang et al., 2023). MXene, with its diverse inherent properties, has demonstrated broad application potential in various fields such as optoelectronics (Tan et al., 2020), sensors (Bhardwaj and Hazra, 2021), electromagnetic shielding (Yun et al., 2020), electromagnetic wave absorption (Ma et al., 2023), and energy storage (Liu L. et al., 2022). Especially in the field of energy storage, MXene has become a highly promising electrode candidate material due to its unique two-dimensional layered structure, tunable interlayer spacing, excellent conductivity, high specific surface area, and adjustable surface functional groups (GaneshKumar et al., 2025). Furthermore, among the numerous electrode materials developed for SCs, MXene stands out for its ability to simultaneously provide double-layer capacitance and pseudocapacitance. By integrating MXene with promising materials such as metal oxides/sulfides and conductive polymers to form hybrid structures, both the energy density and power density of SCs can be enhanced (Boota and Gogotsi, 2018; Meng et al., 2021).

The electrochemical behavior of MXene is not only determined by its intrinsic structure but also influenced by the electrolyte. More importantly, during the preparation and post-processing stages, its key structural features (including interlayer spacing, pore structure, surface end groups, heteroatom doping, defects, and vacancies) can be precisely controlled. Therefore, by understanding the composition of SCs and their different energy storage mechanisms, this review systematically summarizes the application progress of pure MXene nanomaterials in SCs, explores the influence patterns of vacancy doping modification strategies on MXene structure and capacitive performance, and reveals their energy storage mechanisms. This review will be crucial for the rational design of high-performance MXene-based SC devices.

## 2 Research on pure MXene nanomaterials for SCs

It is worth noting that different types of MXene exhibit significant differences in their electrochemical performance. According to reports, Tsyganov et al. (2024) prepared Ti_3_AlC_2_ precursors using the molten salt method and obtained Ti_3_C_2_T_*x*_ under hydrothermal conditions using an HCl+KF mixed etchant. Adhesive-free Ti_3_AlC_2_ thin film electrodes prepared via blade coating exhibited an extremely high mass-specific capacitance of 480 F g^−1^ when tested in 1 M H_2_SO_4_ electrolyte at 25°C under ambient pressure, measured at a scan rate of 1 mV s^−1^ with an electrode mass loading of ∼2 mg cm^−2^. Due to its unique energy storage mechanism, 2D V_4_C_3_T_*x*_ demonstrated stable long-term cycling performance (97.23% capacitance retention after 10,000 cycles in H_2_SO_4_ solution), which has been highlighted as a high-performance material for SCs. The pseudocapacitance of V_4_C_3_T_*x*_ accounts for 37% of the total capacitance (268.5 F g^−1^) in H_2_SO_4_, which is attributed to the stability of vanadium’s oxidation states (+2, +3, +4) (Wang et al., 2019a). Additionally, Ghazaly et al. (2021) investigated the electrochemical behavior of Mo_1.33_C MXene in LiCl electrolyte. The results showed that at a scan rate of 2 mV s^−1^, the volumetric capacity of Mo_1.33_C was 815 F cm^−3^, with a wide operating potential window from −1.2 V to 0.3 V (relative to Ag/AgCl). Further, asymmetric SCs Mo_1.33_C//Mn_*x*_O_*n*_ were constructed, and the device exhibited excellent volumetric performance in 5 M LiCl electrolyte: energy density reached 58 mWh cm^−3^, and maximum power density reached 31 W cm^−3^. After 10,000 cycles at 10 A g^−1^, its capacitance retention rate remained as high as 92%. 2D Mo-based MXenes demonstrate significant potential in energy storage applications, with current research primarily focused on H_2_SO_4_ electrolytes. However, H_2_SO_4_ electrolytes limit the voltage window of symmetric SCs to within 0.9 V and asymmetric devices to within 1.3 V (Ghazaly et al., 2021).

**FIGURE 1:**
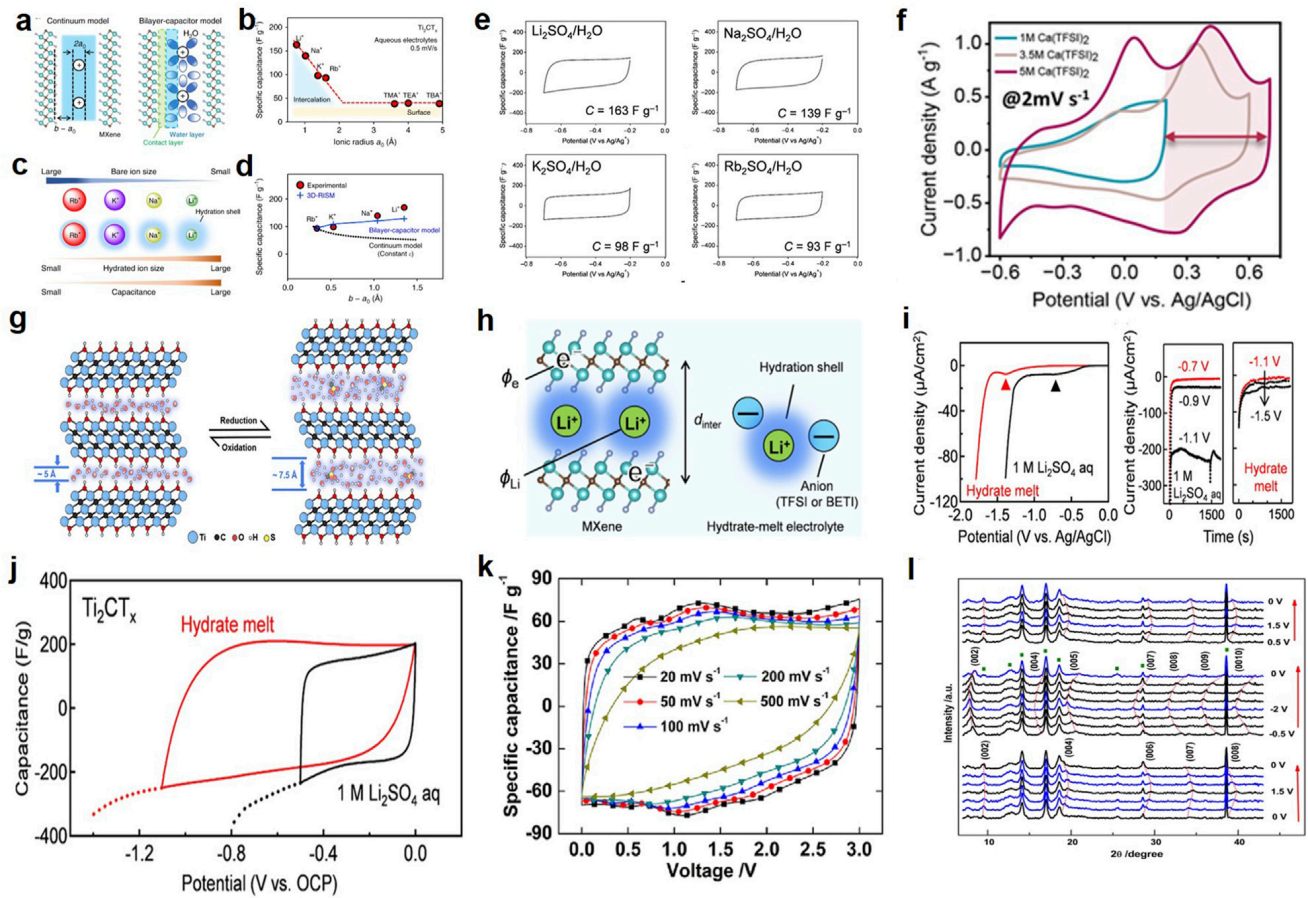
**(a)** Schematic diagram of the continuous model of a micro-gap capacitor and schematic diagram of the double-layer capacitor model (Sugahara et al., 2019). **(b)** Experimental specific capacitance of MXene in aqueous solutions with Li^+^, Na^+^, K^+^, Rb^+^, TMA^+^, TEA^+^, and TBA^+^ electrolytes (Sugahara et al., 2019). **(c)** Order-of-magnitude relationship between bare ion size, hydrated ion size, and observed capacitance values (Sugahara et al., 2019). **(d)** Effect of ion-MXene distance (b−a_0_) on experimental specific capacitance (Sugahara et al., 2019). **(e)** CV curves for MXene with various aqueous electrolytes at 0.5 mV s^−1^. Near-rectangular shape indicates cation intercalation-dominated EDLC behavior (Sugahara et al., 2019). **(f)** CV curves of MXene in Ca(TFSI)_2_ electrolytes at a scan rate of 2 mV s^−1^. Peak distortion confirms pseudocapacitance from surface redox reactions (Yadav and Kurra, 2025). **(g)** Schematic diagram of the charging storage mechanism hypothesis of MXene in non-aqueous electrolytes. Hydrated ion accumulation induces pseudocapacitance at low voltages (Zhao et al., 2019). **(h)** Schematic illustration of Li^+^ intercalation to form an electric double-layer in MXene (Kim et al., 2019). **(i)** Cathodic linear sweep voltammetry of a 1 M Li_2_SO_4_ aqueous electrolyte and a hydrate-melt electrolyte with a Ti electrode at a sweep rate of 0.1 mV s^−1^. Chronoamperometry at various applied potentials vs. Ag/AgCl in a 1 M Li_2_SO_4_ aqueous electrolyte and a hydrate-melt electrolyte with MXene (Kim et al., 2019). **(j)** Cyclic voltammetry curves of MXene with a 1 M Li_2_SO_4_ aqueous electrolyte (black line) and a hydrate-melt electrolyte (red line) at a scan rate of 0.5 mV s^−1^ (Kim et al., 2019). **(k)** CV curves of a 2-electrode Swagelok cell at scan rate from 20 to 500 mV s^−1^ within a voltage window of 3 V (Lin et al., 2016a). **(l)** Electrochemical *in situ* X-ray diffraction study of IL-Ti_3_C_2_T_*x*_ film at various constant potentials (0.5 V steps) in EMI-TFSI electrolyte (Lin et al., 2016b).

MXene electrodes exhibit both capacitive (double-layer capacitance) and pseudocapacitive contributions in electrochemical capacitors. Their primary energy storage mechanism depends on the type of electrolyte. In aqueous SCs, the capacitance of 2D titanium carbides in acidic electrolytes primarily stems from the protonation of H^+^ with MXene oxygen-containing groups (Dall'Agnese et al., 2014). As shown in Figure 1a, hydrated ions are embedded between MXene layers in an aqueous electrolyte system and adsorbed onto the surface via electrostatic forces, thereby primarily forming an EDLC mechanism. Additionally, due to the smaller bare radius of alkali metal cations (Li^+^), which possess higher hydration energy, changes in cations also significantly affect capacitance (Figures 1b–d). The special closed water molecules surrounding the cations shield the external electric field, reducing the potential difference between the ions and the MXene surface, thereby greatly enhancing capacitance (Sugahara et al., 2019). As shown in Figure 1e, in aqueous electrolytes, cation intercalation behavior is prominent, resulting in near-rectangular cyclic voltammetry (CV) curves (Sugahara et al., 2019); in non-aqueous electrolytes, highly reversible redox reactions occur on the surface (Figure 1f), leading to significantly distorted CV curves (Wang et al., 2019b; Ando et al., 2020; Yadav and Kurra, 2025). The use of non-aqueous electrolytes (i.e., organic electrolytes) promotes cation dehydration (Figure 1g), leading to charge transfer between ions and surface groups on two-dimensional titanium carbide (Zhao et al., 2019). Additionally, expanding the operating voltage is another direction for enhancing the energy density of capacitors. As is well known, most organic electrolytes can maintain a wide electrochemical potential window, meaning that the large overpotential between adsorbed ions and MXene surface groups can be overcome (Wang et al., 2019b). Hydrated-melt electrolytes have been applied in aqueous electrolytes to achieve higher operating voltages and energy densities (Kim et al., 2019). In the study by Kim et al. (2019), hydrated Li^+^ ions tightly aggregate on the MXene surface within a wide voltage window (Figure 1h), while the accumulation of hydrated ions induces pseudocapacitance in MXene at low voltages (Figures 1i,j). In addition to the aforementioned electrolytes, Lin et al. (2016a) also constructed SCs using ionic liquid electrolytes and MXene electrodes, achieving a large potential range of 3 V (Figure 1k). Then, the charge storage mechanism was revealed using *in situ* X-ray diffraction. As shown in Figure 1l, unlike the pseudocapacitance generated by the redox reactions of surface functional groups on MXene in aqueous electrolytes, non-boiling ionic liquids cannot induce any redox reactions. The pseudocapacitance of ionic liquid electrolytes, however, originates from the electrostatic attraction between intercalated anions and positively charged MXene sheets during the intercalation process, as well as the steric effects (pillar effects) during the deintercalation process (Lin et al., 2016b).

## 3 Vacancy-doped MXene nanomaterials and their capacitive performances

While pure MXenes exhibit promising charge storage dynamics, their intrinsic capacitance remains constrained by limited active sites and sluggish ion kinetics. To transcend these limitations, vacancy engineering emerges as a paradigm—strategically tailoring atomic-scale defects to reconfigure electronic and ionic landscapes, as systematically deciphered below. Vacancy engineering strategies are classified by elemental identity and synthetic origin: 1) *In-situ* homogeneous metal vacancies (e.g., Ti in Ti_3_C_2_T_*z*_) arise from controlled etching or thermal oxidation, with concentration tunable via etchant severity (Sang et al., 2016); 2) *In-situ* heterogeneous metal vacancies inherit ordered structures from *i*-MAX precursors through selective A-layer etching, enabling zigzag vacancy patterns (Zhou et al., 2025); 3) *In-situ* carbon vacancies form during MXene synthesis via fluorine-free routes or decarbonization, exhibiting high thermodynamic stability (Hu et al., 2017).

### 3.1 *In-situ* homogeneous metal vacancies

**FIGURE 2:**
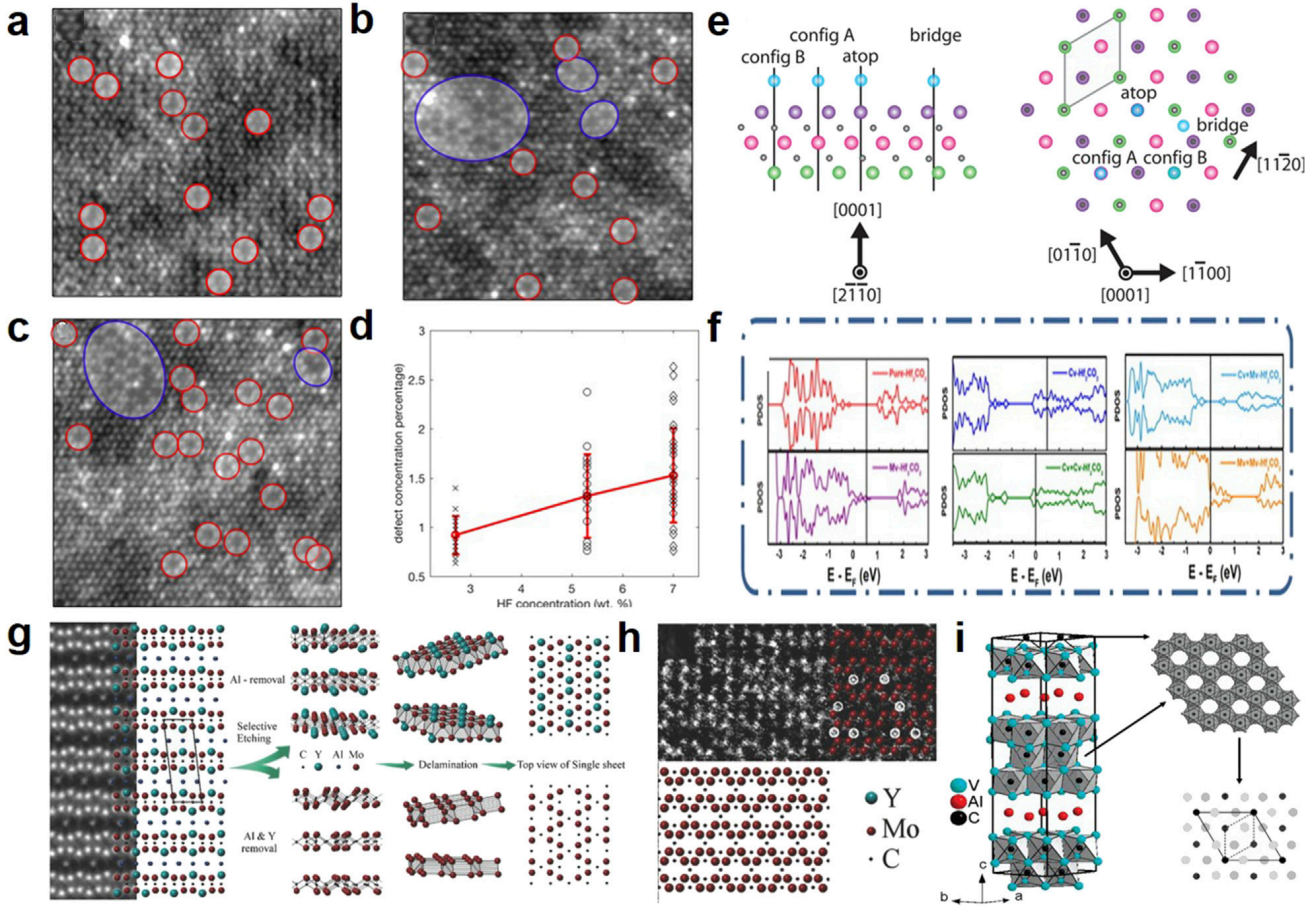
HAADF-STEM images from single-layer Ti_3_C_2_T_*x*_ MXene flakes prepared using etchants with different HF concentrations: **(a)** 2.7 wt.% HF, **(b)** 5.3 wt.% HF, and **(c)** 7 wt.% HF. Single VTi vacancies are indicated by red circles, while vacancy clusters V are shown by blue circles (Sang et al., 2016). **(d)** Scatter plot of defect concentration from images acquired from samples produced using different HF concentrations (Sang et al., 2016). **(e)** Side view and top view of the Ti_3_C_2_T_*x*_ monolayer. The side view shows three titanium layers (green, pink, purple) and two intermediate carbon layers (black), as well as the T_*x*_ positions (blue) distributed along the [2110] zone axis. The top view shows the atomic layers along the [0001] crystal plane axis. The unit cell (approximately 3 Å^10^) is outlined with solid lines (Karlsson et al., 2015). **(f)** The projected density of states (PDOS) diagram of the surface O atoms for Hf_2_CO_2_ with various types of vacancy (Shi et al., 2022). **(g)** (Left) STEM image of (Mo_2/3_Y_1/3_)_2_AlC with corresponding structure model, prior to etching. (Middle) Depending on etching protocol two different structures are obtained; one in which only Al is removed (top) or one in which both Al and Y are removed (bottom). (Right) Top view of corresponding structures obtained (Persson et al., 2018). **(h)** Magnified view of the atomic structure exhibiting the signature zig-zag pattern of vacancy ordered MXene (Persson et al., 2018). **(i)** Schematic diagram of V_2_AlC single crystal and new carbides V_4_AlC_3-*x*_ and V_12_Al_3_C_8_ (Etzkorn et al., 2007).

The direct and *in situ* metal vacancies in MXenes originate from the liquid etching process or the thermal energy from post-heat treatment. As shown in Figures 2a–d, during the liquid etching process, MXene atoms, especially the outer metal (e.g., Ti) atoms, are inevitably removed. Since Ti-deficient MXenes originate from direct contact between the etching solution and the MXene, the concentration of Ti vacancies can be regulated by controlling the concentration of the etching solution (Sang et al., 2016). During heat treatment, C-Ti elongates and fractures, with Ti atoms reacting with -O end groups and separating from TiO_2_ particles (Naguib et al., 2014). Additionally, as shown in Figure 2e, these metal vacancies tend to aggregate and migrate to the edges of MXene layers, forming triangular nanodomains (Karlsson et al., 2015). The introduction of metal vacancies reconfigures the charge distribution around defects, increasing the density of active sites available for ion intercalation (Wang et al., 2022). However, metal vacancies also enhance the material’s catalytic activity (Figure 2f), potentially accelerating electrolyte degradation and significantly limiting its operating voltage window (Shi et al., 2022).

### 3.2 *In-situ* heterogeneous metal vacancies

The remarkable energy storage capabilities of MXene with ordered/disordered vacancies (Mo_1.33_C, W_1.33_C, Nb_1.33_C) have been a focal point of attention. These MXene with ordered vacancies (*i*-MXene) can prepared from the etching of in-plane ordered MAX (*i*-MAX). The structure of *i*-MXene is closely related to the etching process. As shown in Figures 2g,h, Ingemar Persson et al. applied different etching schemes to the parent *i*-MAX phase, namely, varying concentrations of hydrofluoric acid and different etching times. Under harsh etching conditions, compounds with partial removal of Y (Mo_2/3_Y_(1-*x*)/3_)_2_C were formed, and under extended etching times, fully etched Mo_1.33_C was obtained (Persson et al., 2018). When H_2_SO_4_ is chosen as the electrolyte, most MXenes, including Mo_1.33_C, exhibit higher volumetric capacitance because highly reversible redox reactions occur between the -OH groups and H^+^ ions. However, (Mo_2/3_Y_(1-*x*)/3_)_2_C exhibits a completely different trend, showing higher capacitance in KOH electrolyte compared to H_2_SO_4_ electrolyte. The specific composition and arrangement of functional groups on (Mo_2/3_Y_(1-*x*)/3_)_2_C may be the cause of this phenomenon. Through theoretical calculations and experimental verification, it was found that the Mo_1.33_C surface is more prone to forming -F functional groups compared to the original Mo_2_C (Halim et al., 2016; Lind et al., 2017). Additionally, Halim et al. (2018) successfully synthesized and characterized Nb_1.33_C with disordered vacancies. The results indicated that Nb_1.33_C is similar to Nb_2_C, with -O end groups dominating.

To further enhance the electrochemical performance of Mo_1.33_C electrodes, researchers employed two effective strategies: post-etching annealing treatment (Rakhi et al., 2015) and MXene-based hydrogel construction (Jia et al., 2024). The former has been proven to significantly enhance the electrode’s high-rate discharge performance, while the latter is an effective approach to improving its specific capacitance (Ahmed et al., 2020). Additionally, combining Mo_1.33_C with conductive polymers can further improve its capacitance and stability (Qin et al., 2017; El Ghazaly et al., 2021; Ghazaly et al., 2021). For example, to address the brittleness issue of the original Mo_1.33_C film, Leiqiang Qin et al. reported a ‘printed electrode’ (an electrode with high flexibility, light weight, and portability) by mixing Mo_1.33_C with poly(3,4-ethylenedioxythiophene): poly(styrene sulfonate) (PEDOT:PSS) (Qin et al., 2017). SCs constructed using this flexible electrode achieved an energy density of up to 33.2 mWh cm^−3^, a power density of 19,470 mW m^−3^, and a maximum capacitance of 568 F cm^−3^. Considering the synergistic effect between organic and metallic materials, Qin et al. (2019) employed an electrochemical polymerization method to construct interconnected three-dimensional porous polymer-xylene (Mo_1.33_C) composite nanorings. This newly designed electrode can increase the energy density to 20.05 mWh cm^−3^. Additionally, when constructing asymmetric micro-SC with MnO_2_, the operating voltage can be increased to 1.6 V, the areal capacitance can be increased to 69.5 mF cm^−2^, and the energy density can be increased to 250.1 mWh cm^−3^ (Qin et al., 2019).

### 3.3 *In-situ* carbon vacancies

The *in situ* carbon vacancies in MXenes originate from their corresponding MAX phases. As shown in Figure 2i, the V_4_AlC_3-*x*_ compound can be obtained from a Co-containing melt (Etzkorn et al., 2007). Adjacent vacancies in zirconium carbide can enhance its toughness and flexibility (Xie et al., 2016). In addition to conducting experiments, Hu et al. (2017) also used first-principles calculation methods to verify that the conductivity and flexibility of carbon-vacancy Ti_2_CT_2_ are higher than those of perfect Ti_2_CT_2_. Furthermore, unlike metal vacancies in MXene, carbon migration is unrestricted under ambient conditions due to the high migration barrier energy.

## 4 Conclusion and outlook

### 4.1 Conclusion

This review systematically deciphers how atomic-scale vacancy engineering in MXenes—spanning *in situ* defects: homogeneous and heterogeneous metal vacancies, and carbon vacancies—reconfigures electronic, ionic, and interfacial properties to transcend intrinsic capacitive limitations. By establishing clear links between vacancy design (type, density, distribution), electrolyte selection, and device architecture, it provides a foundational roadmap for developing next-generation MXene-based SCs. The work underscores vacancy doping not merely as a materials modification tactic, but as a paradigm-shifting strategy to unlock the theoretical limits of 2D energy storage materials. The specific research content is as follows.

#### 4.1.1 Pure MXene nanomaterial system comparison

Titanium-based MXene (Ti_3_C_2_T_*x*_) performs best in acidic electrolytes (480 F g^−1^); vanadium-based MXene (V_4_C_3_T_*x*_) exhibits a high pseudocapacitance ratio (37%) due to its multi-valent nature (V^2+^/^3+^/^4+^); Mo-based MXene (Mo_1.33_C) achieves a wide voltage window (−1.2 to 0.3 V) and high volumetric capacitance (815 F cm^−3^) in LiCl electrolyte.

#### 4.1.2 Fundamental material design principles

The review establishes that intentional vacancy creation—whether through *in situ* etching of homogeneous metal (e.g., HF concentration-dependent Ti vacancies in Ti_3_C_2_T_*x*_), *in situ* thermal treatment (e.g., C-Ti bond fracture forming TiO_2_), or *in situ* etching of heterogeneous metal (e.g., Mo_1.33_C)—directly enhances MXene’s electrochemical activity. These vacancies can expand active sites for ion adsorption/intercalation, and modulate electronic structure, reducing ion diffusion barriers, and optimize surface chemistry by promoting -O termination over -F.

#### 4.1.3 Structure-property relationship

Vacancy type and distribution: Ordered heterogeneous metal vacancies (e.g., zigzag-patterned Mo_1.33_C) outperform disordered ones in ion-accessible surface area and conductivity due to reduced charge recombination. Electrolyte–electrode synergy: Hydrated ion confinement in MXene interlayers (e.g., Li^+^ in hydrate-melt electrolytes) boosts capacitance by 300% via dielectric constant enhancement. Interlayer spacing control: Vacancies facilitate intercalation of larger ions/organic molecules, widening interlayer gaps and improving EDLC formation kinetics.

### 4.2 Outlook

#### 4.2.1 Machine learning-assisted material screening

Utilizing computational simulation and AI prediction to accelerate the development of novel MXenes (such as double transition metal carbonitrides), optimizing doping sites and vacancy concentrations to achieve synergistic improvements in conductivity and capacitance performance.

#### 4.2.2 Multi-level structural design

Design MXene nanomaterials composed of different elements, such as M-site bimetallic solid solution MXene, X-site N, B solid solution MXene, and medium-high entropy multi-metal solid solution MXene, further achieve vacancy doping and other element doping, optimise the MXene structure, and enhance surface activity.

Combining 3D printing technology to construct gradient-porous MXene electrodes addresses the ion transport bottleneck caused by nanoplate stacking, while a flexible, ultra-thin, all-MXene device design approach achieves high areal capacitance. For example, 3D-printed gradient-porous Ti_3_C_2_T_*x*_ electrodes (Liu G. et al., 2022) mitigate ion transport bottlenecks caused by nanosheet stacking, achieving capacitance retention of 95% at 100 mV s^−1^. In addition, ultra-thin all-MXene micro-supercapacitors (Zhao et al., 2025) demonstrate areal capacitance of 250 mF cm^−2^ via van der Waals self-assembly, highlighting scalable fabrication potential.

#### 4.2.3 Electrolyte engineering innovation

Develop high-voltage/wide-temperature-range electrolytes (such as deep eutectic electrolytes and ionic liquid composite systems) suitable for vacancy-type MXene electrodes to overcome the voltage window limitations of aqueous electrolytes.

#### 4.2.4 Multifunctional integrated device

Develop vacancy-type MXene-based micro-supercapacitor-sensor integrated devices that combine MXene’s electromagnetic shielding and sensing properties to expand its self-powered applications in wearable energy systems.

#### 4.2.5 Sustainable preparation and recycling

Exploring fluorine-free etching processes and closed-loop recycling technologies for vacancy-type MXene waste materials to meet environmental requirements for large-scale production and reduce energy consumption during preparation.

#### 4.2.6 Unresolved challenges and research imperatives

Vacancy stability: Long-term evolution of vacancies during cycling (e.g., aggregation at edges) remains poorly understood. Multi-ion compatibility: Most vacancy studies focus on Li^+^/Na^+^; behavior with multivalent ions (Mg^2+^, Al^3+^) is underexplored. Industrial processing: Vacancy consistency across large-scale MXene production (e.g., roll-to-roll) requires standardized protocols.
